# Challenges in Temperature Measurement in Hot Forging Processes: Impact of Measurement Method Selection on Accuracy and Errors in the Context of Tool Life and Forging Quality

**DOI:** 10.3390/ma18163850

**Published:** 2025-08-17

**Authors:** Marek Hawryluk, Łukasz Dudkiewicz, Jakub Krawczyk, Marta Janik, Marzena Lachowicz, Mateusz Skwarski

**Affiliations:** 1 Department of Metal Forming, Welding and Metrology, Wroclaw University of Science and Technology, Wybrzeże Wyspiańskiego 27, 50-370 Wroclaw, Poland; ldudkiewicz@schraner.pl (Ł.D.); krawczyk.jakub@outlook.com (J.K.); martaczubak@wp.pl (M.J.); marzena.lachowicz@pwr.edu.pl (M.L.); mateusz.skwarski@pwr.edu.pl (M.S.); 2 Center for Materials Science and Metal Forming, Lukasiewicza 5 Street, 50-371 Wroclaw, Poland; 3 Schraner Polska Sp. z o.o., Lotnicza 21g Street, 99-100 Łęczyca, Poland

**Keywords:** temperature field determination, problems of temperature measurements, die forging at elevated temperature, thermal measurement devices

## Abstract

This study investigates the influence of temperature measurement accuracy on tool failure mechanisms in industrial hot forging processes. Challenges related to extreme operational conditions, including high temperatures, limited access to measurement surfaces, and optical interferences, significantly hinder reliable data acquisition. Thermal imaging, pyrometry, thermocouples, and finite element modeling were employed to characterize temperature distributions in forging tools and billets. Analysis of multi-stage forging of stainless steel valve forgings revealed significant discrepancies between induction heater settings and actual billet surface temperatures, measured by thermal imaging. This thermal non-uniformity led to localized underheating and insufficient dissolution of hard inclusions, confirmed by dilatometric tests, resulting in billet jamming and premature tool failure. In slender bolt-type forgings, excessive or improperly controlled billet temperatures increased adhesion between the forging and tool surface, causing process resistance, billet sticking, and accelerated tool degradation. Additional challenges were noted in tool preheating, where non-uniform heating and inaccurate temperature assessment compromised early tool performance. Measurement errors associated with thermal imaging, particularly due to thermal reflections in robotic gripper monitoring, led to overestimated temperatures and overheating of gripping elements, impairing forging manipulation accuracy. The results emphasize that effective temperature measurement management, including cross-validation of methods, is crucial for assessing tool condition, enhancing process reliability, and preventing premature failures in hot forging operations.

## 1. Introduction

Among many manufacturing technologies, die forging at elevated temperatures is widely used in the production of various elements for the machinery, agricultural, mining, and, above all, automotive and aviation industries, whose products are expected to have high operational properties and operational reliability [1,2]. Currently, the main directions of development of the forging industry focus on increasing the quality of forgings [3] and increasing the efficiency of forging processes through their automation and robotization [4] and the use of advanced, often mechatronic production monitoring systems in order to reduce unit production costs [5]. In this respect, the aim is to increase the efficiency of the entire shaping process, as well as to increase the durability of the shaping tools used [6,7] and, above all, to eliminate defects in forgings that reduce production efficiency [8,9]. Currently, a number of non-destructive methods and tools are used to comprehensively analyze and optimize the entire forging process, including the so-called engineering works, such as CAD/CAE/CAM [10,11,12], as well as other IT tools, in particular, physical and numerical modeling and simulations. Of these, numerical modeling based on FEM/FVM [2] is currently the most frequently used method for the analysis and optimization of many manufacturing processes. Still, other non-destructive measurement methods, very often used in industry, including forging processes, include thermal imaging and, in particular, non-contact temperature measurements using thermal imaging cameras. Temperature measurements using non-contact methods, thanks to significant technical progress in the field of electronics, are increasingly used in industrial practice [13,14]. The main advantage is much easier operation, increased functionality of this type of device, and the ability to visualize the results on an ongoing basis. The disadvantage in the case of non-contact measurements is the measurement of the temperature distribution just on the surface, not inside [15]. It should also be added that temperature measurements using thermal imaging are indispensable in the case of, for example, verification or determination of initial boundary conditions in numerical modeling [16]. The inevitable confounding factors resulting from non-contact temperature measurement must also be taken into account [17]. In the case of hot die forging processes, apart from ensuring the appropriate (in accordance with the given technology) heating temperature of the charge material, which, for steel forgings, is usually above 1100 °C, as well as the appropriate working temperature of the forging tools, a key aspect is also temperature control and correct and continuous temperature measurement during forging. The correct temperature of the charge material and the working temperature of the tools are a guarantee of success, resulting in high quality forgings, on the one hand, and durability of the forging equipment [18]. The heating of the steel charge material is usually carried out by an induction furnace due to its high efficiency and relatively high accuracy. In the case of forgings made of aluminum alloys, the charge is heated in electric furnaces [19]. However, the temperature measurement of the charge material itself is most often carried out using pyrometers and does not pose a major problem. However, ensuring a stable temperature of the forging tools and its optimal and verified measurement using selected measurement methods is a certain challenge [20]. During forging and hot forging, material comes into contact with the tool, and there is a risk of overheating or cracking due to uneven and rapid heating of the tool material, as well as excessive cooling during lubrication after forging [21]. It is important to consider the thermal balance associated with the heat generated during the deformation of the forging material (as the work of plastic deformation is converted into heat) and the heat produced from friction, along with the cooling effect of lubrication on the tools [22,23]. This balance is influenced by the ratio of the tool’s volume/mass to the input material. Incorrect proportions or improper selection can lead to overheating or cooling of the tool during the production cycle, potentially resulting in premature wear [24]. Therefore, particularly concerning the durability of tools, it is crucial to ensure proper preheating before the forging process and to maintain stable tribological conditions that provide thermal balance during forging, along with continuous temperature monitoring [25]. Currently, a good direction of research in the area of temperature change analysis is also the use of numerical modeling, which allows for a comprehensive analysis and determination of the temperature field in die forging processes in a relatively fast and accurate way [26,27]. The innovative nature of the research presented in this article is highlighted by its applicability across various facets of the die forging industry during measurement and analysis of temperature. Challenging operational conditions and economic constraints within the forging industry have hindered the adoption of such methods as supportive tools in forging processes. However, with ongoing technical and technological advancements, it is now feasible to implement more advanced temperature measurements methods, often adapted from other fields, even in heavy industries, like die forging processes conducted at elevated temperatures.

Based on many years of experience in hot die forging processes, the authors conducted a comprehensive analysis of the influence of temperature measurement accuracy on tool failure mechanisms, relying on specific industrial cases. This approach combines experimental research with numerical modeling and multi-method measurement verification, which allowed for the identification of key relationships between temperature distribution and the occurrence of adhesion phenomena and premature tool wear. This study also includes practical recommendations, such as optimization of heating time, which can contribute to improving tool durability and the reliability of forging processes.

The aim of this study was to determine the influence of temperature measurement accuracy on tool failure mechanisms in hot forging processes, considering the properties of the billet materials and the effectiveness of various measurement methods. The relationships between temperature distribution, heating level, and billet hardness, and the occurrence of phenomena such as adhesion and premature tool wear were analyzed.

## 2. Materials and Methods

**Figure 1:**
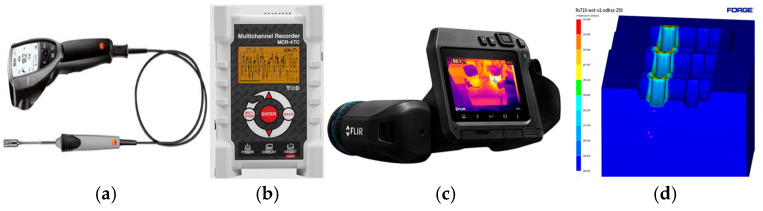
Images of the measurement devices utilized in the temperature variation tests include the following: (**a**) a Testo 830_T2 laser pyrometer (Testo SE & Co. KGaA, 79822 Titisee-Neustadt, Niemcy) equipped with a thermocouple, (**b**) an MCR-type temperature recorder (T&D Corporation, 390-0853 Matsumoto, Japonia) also using a “K”-type thermocouple, (**c**) a Flir T540 thermal imaging camera (Teledyne FLIR, 97070 Wilsonville, OR, USA), and (**d**) sample temperature distributions derived from numerical modeling using Forge 3.0NxT software (Transvalor S.A., Biot 06904 Sophia Antipolis cedex, France).

The subject of this study was the use of various temperature measurement methods and devices to identify the most effective and reliable approach for monitoring and analyzing temperature changes in billets, forging tools, and other components involved in industrial hot forging processes. Two groups of forging processes were analyzed, differing in the billet material type and the friction model adopted in numerical simulations. The first group included forging processes of billets made of carbon steel, primarily 42CrMo4, which is widely used in machine and tool components due to its good strength and thermal conductivity. The second group involved processes where the billet material was NC3015, a heat-resistant chromium–nickel steel used in high-temperature applications. To carry out the scientific research and meet the research objectives, a wide array of methods and measurement tools was utilized (Figure 1).

Three temperature measurement methods were used in this study:

Testo 830-T2 laser pyrometer with a K-type thermocouple (accuracy ±1.5 °C);
MCR-type temperature recorder with K-type thermocouples (sampling rate 1 Hz);
Flir T540 thermal imaging camera (spectral range 7.5–14 µm; accuracy ±2 °C).

Measurements were performed in three key locations: on the billet surface before deformation (1), in the billet–tool contact zone during forging (2), and in the flash area after the process (3). Each measurement was repeated at least five times per trial, and the results were verified based on the analysis of 5 to 10 forging series in each process group. This ensured sufficient repeatability and reliability of the measurement data.

Additionally, numerical modeling was performed using the Forge 3.0NxT computational package, as well as Marc Mentat software, ver.18 (MSC Software, Munich, 81829, Germany). In the simulations (first group), Coulomb’s friction model was applied with a friction coefficient of μ = 0.3. Due to the specific nature of the billet–tool contact, Tresca’s friction model was used in the numerical simulations, with a friction factor of m = 0.4.

In all the analyzed cases, forging tools in the numerical models were assumed to be made of tool steel 1.2343, which is commonly used in hot forming applications. However, in real industrial conditions, tool steels such as W360, WLV, or 1.2344 may also be used. Since the differences in chemical composition and mechanical/thermal properties between these materials are relatively small, the use of 1.2343 in simulations is justified and does not significantly affect the reliability of the results. In Table 1 is presented the chemical composition of the analyzed materials (both for charge and tool).

For each process group, 5 to 10 forging trials were performed using new billets. The billet heating temperatures were as follows: for carbon steel, approx. 1150 °C; for NC3015, approx. 1120–1140 °C. The tools were preheated to 250–400 °C. Taking into account realistic boundary conditions, the initial temperatures of the billet and tools and heat transfer through conduction, convection, and radiation were friction modeled according to the selected approach (Coulomb or Tresca).

Geometric models were created using tetrahedral and hexahedral elements, which allowed for accurate representation of the billet and tool shapes, as well as the complex thermomechanical phenomena occurring during the process.

Detailed information on the numerical modeling, technological assumptions, and experimental procedure is provided in the subsequent subsections.

## 3. Research and Results Discussion

### 3.1. Thermal Imaging Tests for Analyzing Temperature Changes

**Figure 2:**
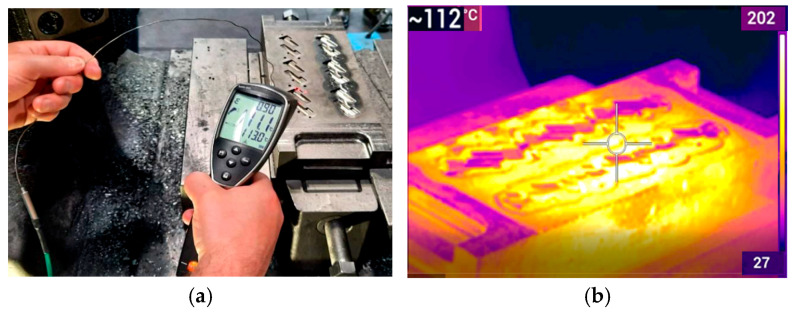
Measurements and verification of forging tools temperature by use: (**a**) pyrometer with thermocouple (contact measurement), (**b**) thermovision camera.

Thermography is the safest way to measure the temperature fields of both the workpiece and the tool during forging processes, as other methods encounter difficulties due to high temperatures and pose risks to operators. Precise determination of the emissivity of the material and calibration of the measurement range enable the detection of precise temperature zones. Based on the temperature measurement results, it is possible to analyze and conclude what phenomena and changes occur during the process. Unfortunately, it sometimes happens that in some cases, measuring the temperature of a given object is difficult or even impossible. The use of a thermal imaging camera is particularly useful when it is not possible to perform contact measurements, which are usually more accurate. Thermal imaging measurements not only provide non-contact and safe method of temperature monitoring but also enable the visualization of complex thermal gradients and transient phenomena that are difficult to detect using traditional techniques. This analytical capability makes thermal imaging especially useful in diagnosing uneven heating, local overheating, or thermal losses in industrial forging processes. Of course, you should also be aware of errors with such a device, which most often result from inappropriate settings of the emissivity factor, reflections, and other process disturbances. However, an experienced researcher is able to deal with all these problems and is fully aware of what and how to measure and what the measurement error may be. The authors also have extensive experience in the use of thermal imaging to study temperature changes. In the case of measurements using non-contact measuring devices, it is very important to set the appropriate value of the emissivity coefficient and avoid reflections and other sources of heat, especially at higher temperatures. The emissivity coefficient is especially crucial because the accuracy of the measurement depends on its value. It is normally set to a value in the range of 0.9–0.95. However, in the case of forging processes, which are very dynamic and characterized by rapid temperature changes, setting the appropriate emissivity coefficient is difficult. Therefore, most often, to verify temperature measurements using a thermal imaging camera, the contact method is used, e.g., a pyrometer with a thermocouple (Figure 2a,b), based on which the appropriate emissivity value can be easily determined. Based on the authors’ experience, in the case of hot forging, the emissivity value for non-contact devices was set at 0.8–0.9, which, based on the results obtained, gave the most reliable results.

**Figure 3:**
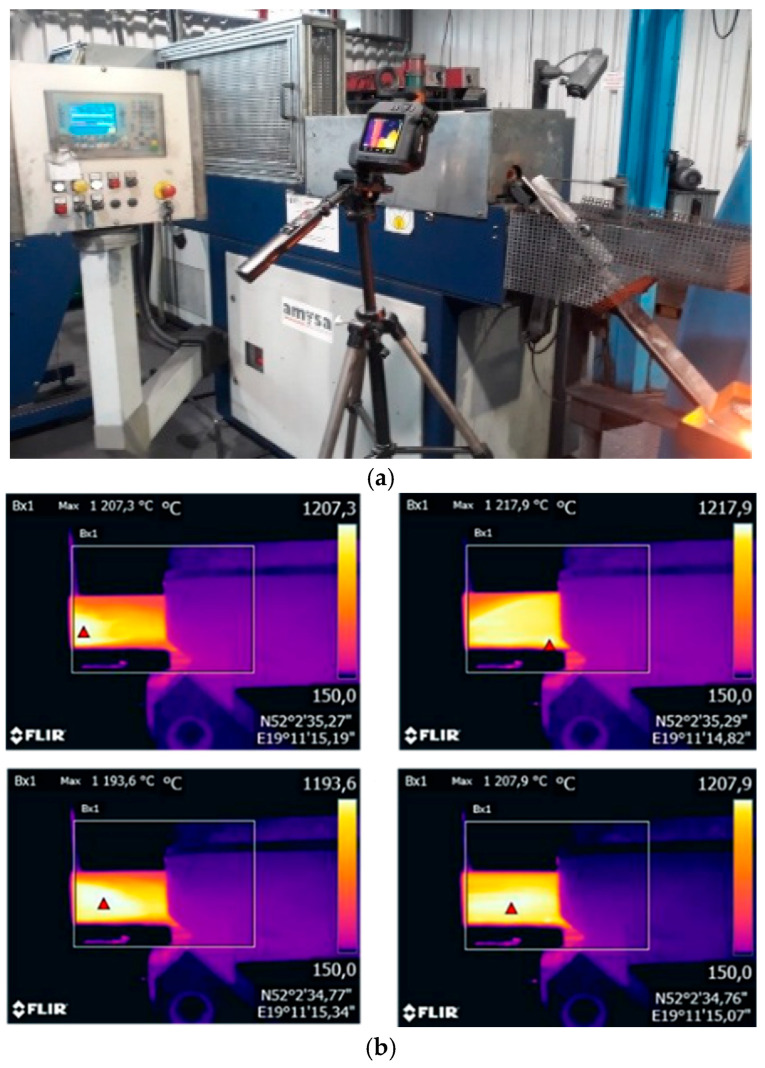
A view of (**a**) the research and measurement station for measurement and analysis of temperature changes of the input material and (**b**) thermograms with the temperature field distributions for selected charges.

The results of the tests using contact and non-contact devices shown in Figure 2 showed good agreement in this case, with the emissivity set at 0.85, and thus provided the basis for temperature measurements using a thermal imaging camera. The thermal imaging camera (verified based on previous calibration using a thermocouple and setting the emissivity coefficient) was then used to measure and analyze the temperature field distributions for the heated input material in the form of a 16MnCrS5 (1.7131 according to DIN standard [28]) steel cylinder, which were recorded at the initial stage of the production process, for stabilized operating conditions, in order to determine the correctness and stability of heating. Figure 3 shows the measuring station with a thermovision camera and thermograms with the temperature field distributions of several selected preforms just after leaving the induction heater.

**Figure 4:**
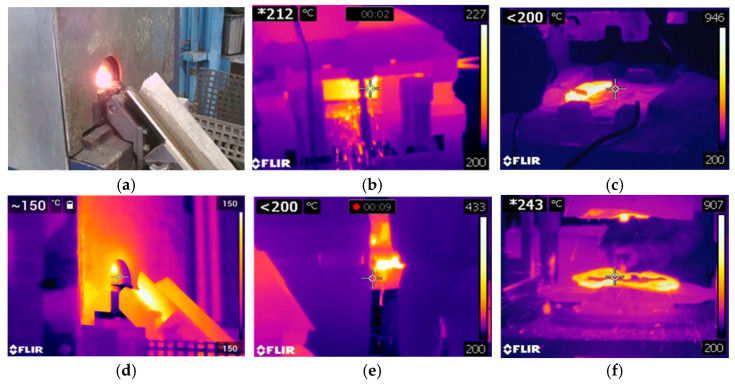
Analysis of temperature changes from selected operations of the multi-stage process of producing a lever-type forging for a motorcycle: (**a**) photo of the heated charge leaving the heater, (**b**) thermogram for this situation, (**c**) de-calculation of the charge material after heating, (**d**) rolling the charge onto a blank forging, (**e**) die forging, (**f**) flash trimming process, * indicates the exact temperature value at the point marked on the thermogram (at the crosshair location).

The temperatures obtained using the thermal imaging camera (Figure 4c) during the measurements of successive heated billets (charge) were compared with the maximum temperature indicated by the heater (measured using a pyrometer permanently installed at the device’s outlet). All the measurements taken are presented in Table 2.

The measured temperatures differ from the assumed ones but are still within the permissible range, i.e., above the minimum hot forming temperature of 1.7131 steel (1100 °C, above which recrystallization occurs in the structure) and below the maximum forming temperature (1300 °C, above which an undesirable liquid phase could appear in the structure). Figure 4 shows the results of thermal imaging tests for an example technological sequence of the hot forging process in order to assess the impact of high temperatures on the material of tools used in the multi-operation process of producing a motorcycle lever forging [23].

The test results presented in Figure 4, mainly in the form of thermograms from selected stages of the technology, allow for a relatively easy analysis of temperature changes in the tools used in these operations and, on this basis, the determination of places where high temperatures may cause, for example, local tempering of the material and a decrease in durability, and may also cause premature wear.

**Figure 5:**
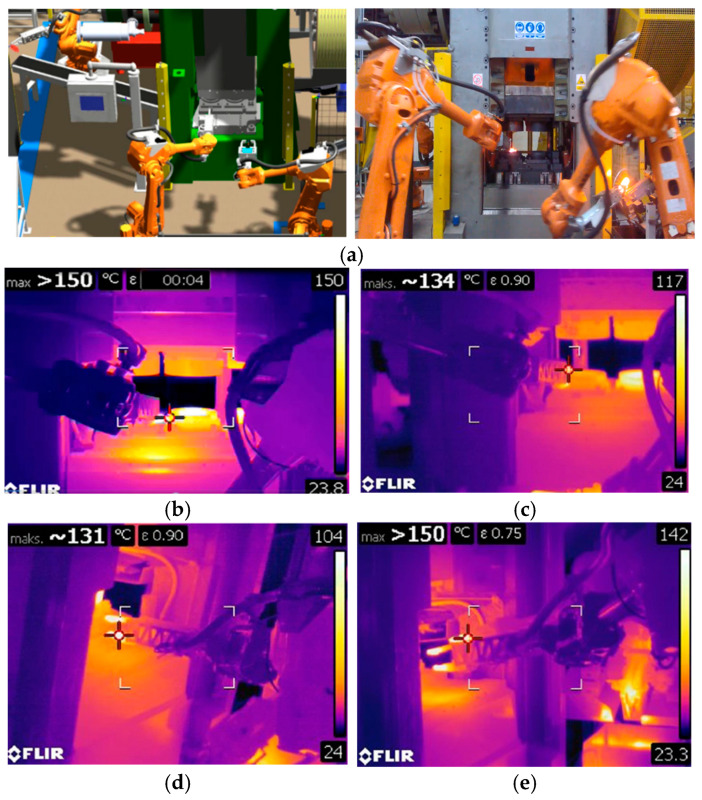
Test results: (**a**) the example photo from simulation of the robotic station in Robot Studio (left) and photo from real robotic industrial forging process (left), (**b**) thermograms with temperature distributions when transferring forgings from the preliminary forging operation to the finishing forging operation, (**c**) close-up of the analyzed area in (**b**), (**d**) thermogram for the second robot, (**e**) detailed analysis of temperature changes on the gripper.

A different use of a thermal imaging camera was also used to assess the effect of temperature changes on robot grippers during the hot die forging process of a forked-type forging. Figure 5a shows a photo from the simulation of the forging process, in particular, the analysis and research of the robot movement trajectories carried out in the Robot Studio program, ver. 2023.1 (RobotStudio, ABB, 721 78 Västerås, Sweden). Figure 5b shows a thermogram showing a similar moment of forging, in which a smaller range of maximum temperature during the forging process was deliberately set so as to analyze the temperature for the grippers themselves more precisely. Based on observations, it can be seen that the parts of the gripper that do not have direct contact with the hot material heat up to a temperature of over 120 °C.

However, those parts that are in direct contact have a temperature of about 150 °C. A more thorough analysis of this situation (Figure 5c) shows that the maximum temperature for the gripping part contacting the forging is 134 °C. In turn, Figure 5d shows a thermal image of the second manipulator gripping and transferring the charge material. As can be seen, in this case, the maximum temperature is similar to that previously observed for a manipulator carrying a hot forging and is approximately 130 °C. As can be seen in Figure 5e (left of the viewfinder), the gripper temperature after several minutes of operation reached a temperature exceeding 150 °C. The analyses performed showed that the variability and temperature range were determined to be safe for the gripper material used, where, for the assumed steel grade, tempering occurs after exceeding the minimum temperature of 450 °C. According to the assumptions, the temperature of the working elements decreased proportionally with the increase in the distance from the heat-affected zone, where at the height of the actuator driving the grippers, it did not exceed 30–45 °C, which was considered consistent with the working conditions [29]. Therefore, the thermal resistance leads to the repeatability of gripping and shifting the manipulator.

**Figure 6:**
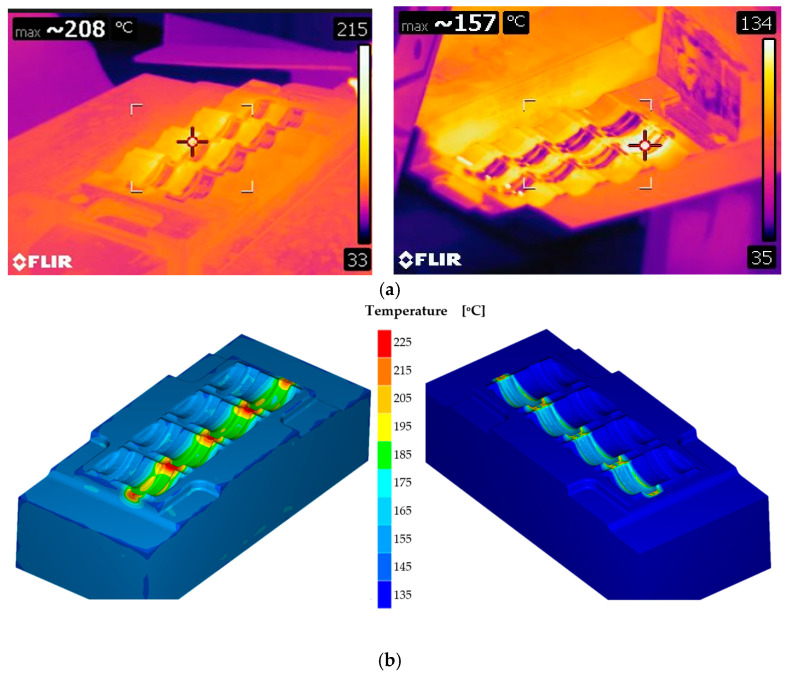
Results of temperature measurements: (**a**) examples of thermovision measurements for lower and upper die, (**b**) FEM results of temperature.

Thermal vision measurements were used to analyze the impact of tribological conditions on the adhesion and curvature of forged elements—window bolt forgings produced in multiple systems (Figure 6).

Based on the analyses carried out using thermal imaging cameras, it was possible to identify areas of tools for specific blanks for which differences in temperature distributions can be noticed, which, in turn, are caused by the manual lubrication system, which causes different temperature distributions in individual forging cavities [26].

**Figure 7:**
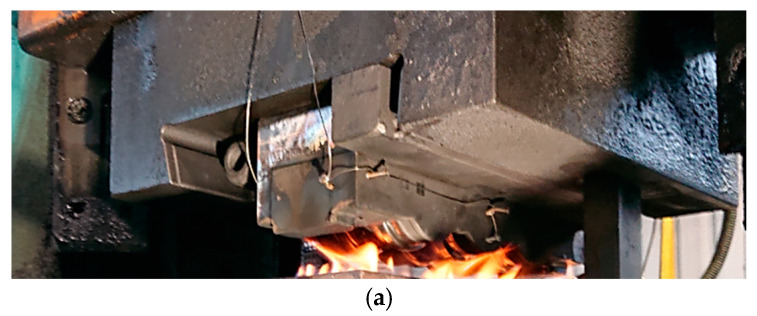

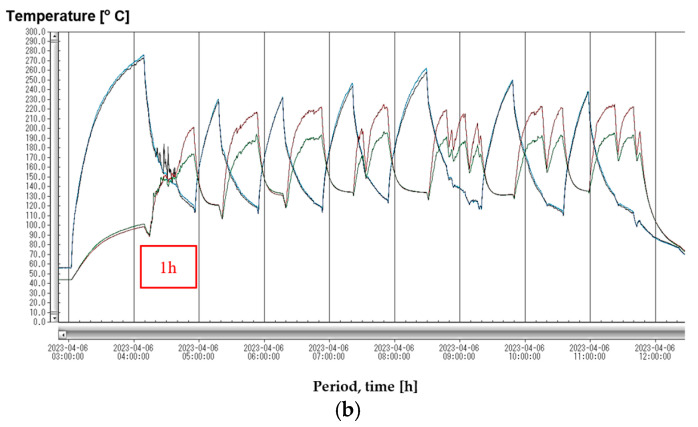
The view of (**a**) upper die forging assembled on hydraulic hammer with thermocouples during heat-up by gas burner, (**b**) results of temperature measurements into die by thermocouples (red—roughing pass, lower tool; green—finishing impression, lower tool; blue—roughing pass, upper tool; black—finishing impression, upper tool).

The study of temperature variations in the tools (upper and lower dies) during the initiation and continuation of the process involved prolonged measurements and recording of temperature changes using thermocouple sensors. The research was conducted to analyze the potential cooling of the dies due to insufficient heat energy being supplied during forging, which was attributed to the relatively small volume of the forging compared to the die block. Based on the developed technical and technological solution, appropriate holes were made using an erosion drill, into which thermocouples were mounted. Data was recorded using a portable MCR-4TC recorder. Measurements were taken using K-type thermocouples, whose ends were mounted in blind holes located under the central cavity of the rough and finishing dies, along the symmetry axis of the given die, approximately 10 mm below the die. Figure 7 shows the forging die after being mounted on the hammer during heating with a gas burner, as well as history results from measurements during one shift.

Figure 7b presents the results of measurements for a few production cycles, consisting of (about one hour) heating of the forging tools for the forging process, the forging process, reheating of the forging dies, and so on. During analysis of the temperature profiles, it can be observed that the temperature of the lower forging tool stabilizes during the forging process, reaching approximately 200 °C for the preliminary die and around 170 °C for the finishing die. Meanwhile, the temperature of the upper forging tool decreases throughout the process. The upper tool initially reaches temperatures of 240–260 °C in the rough and finishing impressions, but during the process, it drops to about 135–145 °C. After the final working temperature, the upper tool’s temperature decreases to 240 °C in both dies. The results confirm the assumption that the operating temperature of both tools decreases during the process. Consequently, it was decided to use a separator between the forging tool and the anvil block of the forging aggregate. After a detailed analysis, a 4 mm thick separator made of Duro Best 300 was selected. This material is a composite based on epoxy resin, with a declared working temperature of up to 300 °C, a compressive strength of 500 MPa, and a thermal conductivity coefficient of 0.23 W/m K, which is about several dozen times lower than that of hard work tool steel.

### 3.2. Numerical Modeling of Temperature Field Distributions

**Figure 8:**
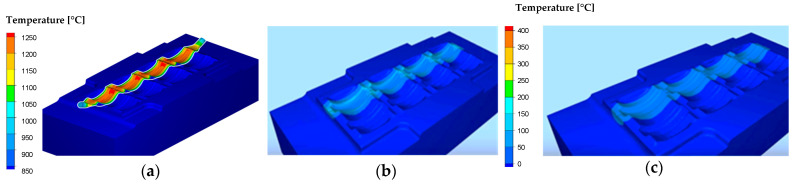
The FEM results: comparison of the influence of the initial heating temperature of the forging dies on the heat transfer by the forging during pre-die forging. (**a**) Distribution of the temperature field in the forging after the forging operation (temperature scale for the forging), (**b**) initial temperature of the dies (100 °C), (**c**) initial temperature of die (250 °C).

Numerical simulations related to determining temperature changes in forging processes are an important aspect of the use of FEM, as well as non-destructive research and measurement methods. Often, this is the only way to determine the temperature field distribution due to the lack of another option, i.e., direct measurement (contact/non-contact), as is the case with forging processes. On the other hand, it should be mentioned that the determined temperature distributions often constitute important boundary initial data for various analyses and numerical modeling. The application of numerical simulations to analyze temperature changes was used to assess the impact of the initial heating temperature of the dies for two values, 100 °C and 250 °C, during the forging operation of clamp-type forgings in a triple system on the heat transfer before the forging (Figure 8).

**Figure 9:**
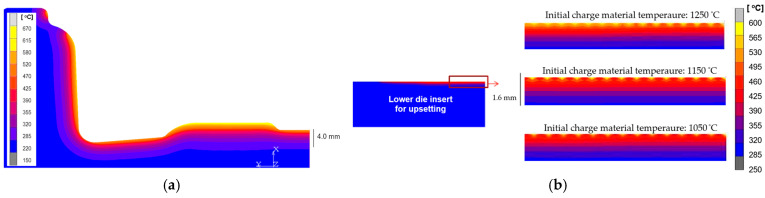
The FEM simulation results: (**a**) distribution of the temperature field in the lower die insert (initial temperature of tools: 250 °C; forgings: 1100 °C), (**b**) influence of changes in the temperature of the charge material on the distribution of the temperature field in the tool.

To better illustrate the impact of initial die temperature on thermal changes during forging, Figure 8a presents the temperature distribution in the forging after the preliminary operation, using a scale set for the forging itself. As shown, a lower die temperature of 100 °C (Figure 8b) results in significantly greater heat absorption from the forging compared to a higher die temperature of 250 °C (Figure 8c), despite the same forging temperature of 1200 °C. This effect is especially noticeable in the bridge areas, where intense material flow occurs—here, the groove temperature reaches ~260 °C for the hotter die but only ~140 °C for the cooler one. Figure 9a shows the numerical simulation results of the temperature field on the upper and lower die inserts immediately after forging [30].

It can be seen that the heat-affected zone reaches a depth of approximately 4 mm, and the highest temperature value reaches over 600 °C (the initial temperature of the charge material was 1150 °C). Such results allow determining the distribution of the temperature field, which plays a very important role, especially in the surface layer of the tool, because its changes during the process will determine the intensity of destructive phenomena that reduce the durability of the tools [22]. It should be emphasized that this is difficult to achieve even with the use of an advanced thermal imaging camera. In turn, Figure 9b shows the impact of changing the temperature of the feed material on the distribution of the temperature field in the matrix insert used in the first (upsetting) forging operation. It is worth emphasizing here that obtaining correct results related to temperature distributions in the case of numerical modeling depends largely on the experience and knowledge of the use of computational packages by users because many factors influence the quality of the obtained results. The obtained temperature field distributions in the tool (average element size 0.2 mm) with an initial temperature of 500 °C for various preform temperatures (from 1050 to 1250 °C) indicate that in the case of modeling, the size of the heat-affected zone does not change, but the temperature on the surface changes significantly (from 530 to approximately 600 °C). In the case of numerical modeling, in terms of the accuracy of the results, the size of the finite elements used is crucial because on the one hand, a large number of small elements provides greater accuracy, but on the other hand, it also significantly increases the time of numerical calculations.

**Figure 10:**
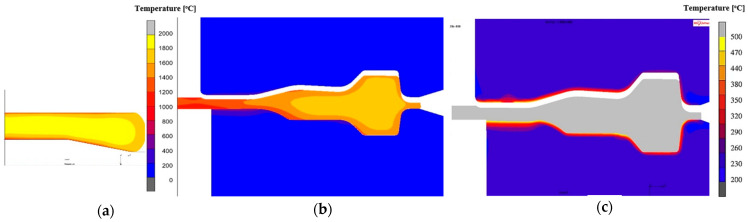
Temperature distribution for (**a**) forging in the final phase of the first forging operation, (**b**) third forging operation, (**c**) temperature field distributions for die inserts after the third forging operation.

Numerical modeling using the Marc Mentat program was used to analyze the temperatures for the front wheel forging and their effect on the temperature field distribution for the die inserts. Figure 10a,b show the temperature distributions after the first and third operation. It can be seen that the temperatures in the forgings are similar to the preform temperature (1150 °C). However, due to the cooler tools on the contact surface, the temperature is much lower (even by 350 °C in the case of the first operation and 200 °C in the case of the third operation). On the other hand, the high temperature inside the forgings is the result of large plastic deformations and the conversion of the plastic deformation work into heat. The numerical analysis of shaping tools is very interesting in the context of durability. Currently available computational packages have in their architecture more and more various functions that allow for a detailed analysis of the condition of forging tools.

**Figure 11:**
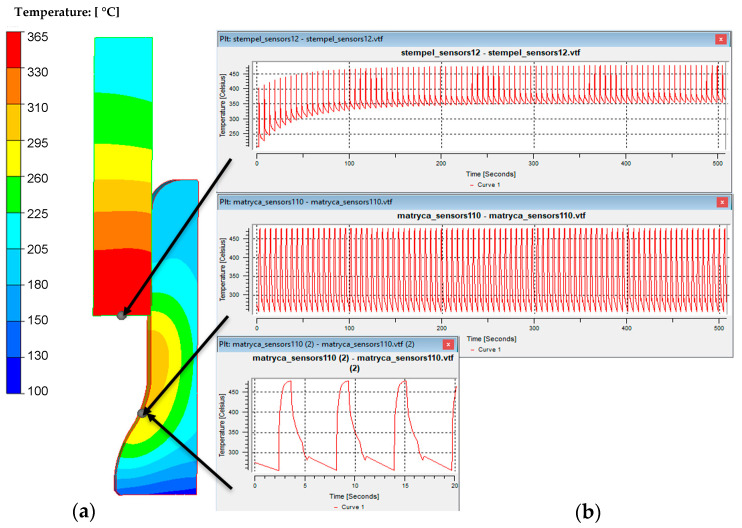
The FEM results: (**a**) temperature field distributions for tools (steady state), (**b**) temperature courses in 500 consecutive forging cycles for selected points on the punch and die.

Figure 10c shows the temperature field distributions in the tools used in the second operation, in the final phase of deformation. The maximum temperature (over 600 °C) occurs on the central surface of the lower die insert (the place of contact of the preform from the first operation) and on the radii of the impression (places of intensive material flow—large friction path). Such a high temperature in these areas can cause the occurrence of local plastic deformation (softening of the tool material). As part of numerous works [30], “steady-state” numerical simulations were also carried out in the Forge program for the process of extruding an engine valve forging from NC3015 steel (Figure 11). The modeling was conducted to identify the temperature profiles and values, as well as to determine the number of forging cycles needed for the process to reach a steady state. This steady state is defined as the point where the temperature difference between two consecutive forging cycles is less than 0.3 °C.

**Figure 12:**
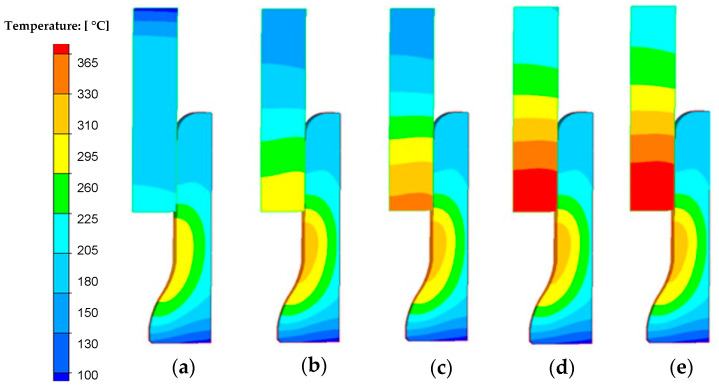
FEM results with temperature distribution after (**a**) 1 cycle, (**b**) 5 cycles, (**c**) 10 cycles, (**d**) 50 cycles, (**e**) 90 cycles of the first hot forging operation of the exhaust valve.

Based on the presented results (Figure 11), it can be observed that in the case of the analyzed process, a stable state related to temperature changes, especially on the punch, can be observed for approximately the 90th forging cycle. Figure 12 shows the results with the temperature field distributions for several selected initial forging cycles.

A careful analysis of Figure 11 and Figure 12 shows that the temperature on the die stabilizes after several forging cycles in the first operation. The temperature on the punch takes longer to stabilize (continuous increase is visible for approximately 200 s), which is probably related to the lack of cooling of the punches in the considered forging process. It should also be noted that tests using FEM are usually carried out for one cycle, most often the first one, which does not allow for a full analysis of, for example, changes in temperature in tools over a longer period of operation. However, modeling a larger number of cycles in FEM is problematic. Moreover, the initial states in industrial processes are unstable, variable, and unsteady, which makes it difficult to interpret the results. However, the modeling results for a large number of forging cycles, as is the case in a typical industrial forging process, are much more interesting.

### 3.3. Application of Different Methods of Temperature Measurements to Solve Problem of Premature Wear of Dies Used in the Production of Engine Valve Forgings

**Figure 13:**
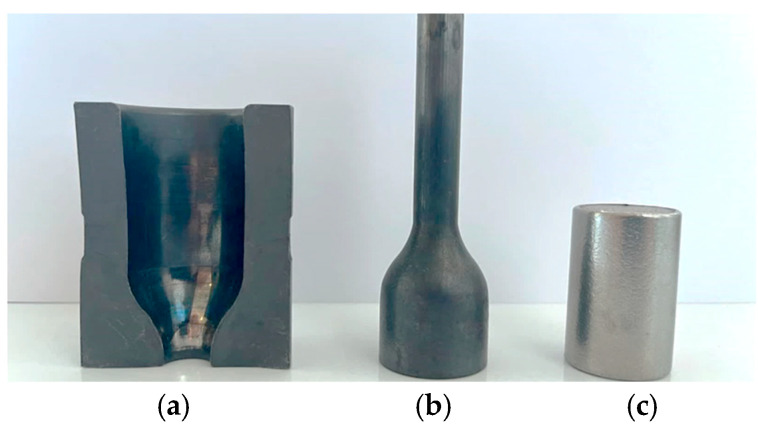
A view of (**a**) a die’s cross-section, (**b**) the forging after extrusion, (**c**) the charge material.

A combination of the selected temperature measurement methods presented and characterized above was used in conjunction with other studies to analyze and solve the problem of premature wear of forging tools in the two-stage forging process of a valve forging. A detailed analysis was performed of forging tools (dies used in I operation) of hot forward extrusion of a long leg tipped with a pre-formed element (Figure 13), for which, in the case of insufficient dissolution of the hard inclusions and fractions in the heated charge material, blocking or fast damage of such a tool took place [31].

**Figure 14:**
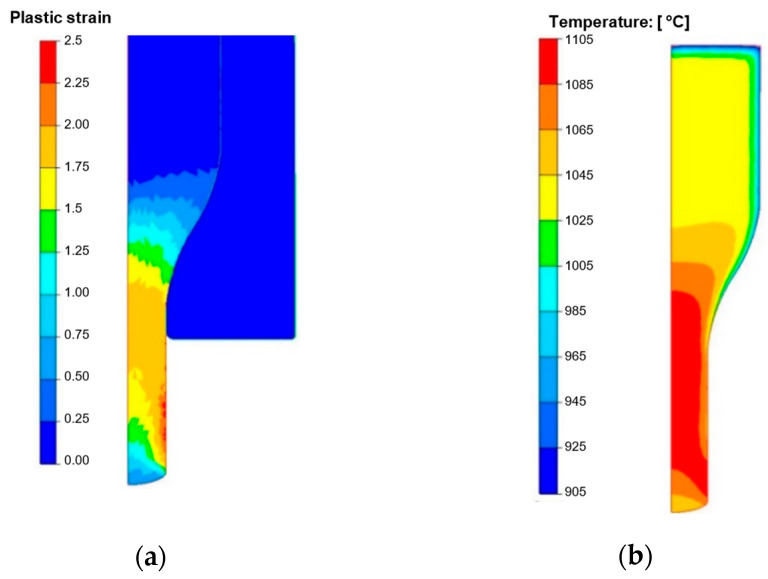
The final phase of forging with distribution of (**a**) plastic strains, (**b**) temperature of forging after first operation.

In order to obtain the required material temperature, induction heating is applied at a temperature of 1040–1060 °C before the forging. The microstructure of the preforms after induction heating characterizes in the presence of polygonal austenite grains with a much bigger grain than the as-delivered material. It is very important that the M_23_C_6_-type carbides undergo total dissolution as a result of such heating because this will provide the heated charge material with the proper hardness, at the level of 190–220 HV. For this reason, to solve this problem, numerical modeling was applied with the use of the Forge 3.0 NxT program. The numerical simulations of the forging process were performed for the operation of coextrusion for two options. The first for the standard process was an input material temperature of 1050 °C, for which the hardness was assumed the 210 HV. For the second option, we assumed an input material temperature of 100 °C lower, i.e., 950 °C, which placed the charge material hardness at the level of 290 HV. The calculations were performed with the aim to compare the most important key parameters, which decide about the tool life (von Misses pressures, forging forces, etc.). The following boundary and initial conditions were assumed, based on a standard process: nominal temperatures: input material—1050 °C, die and punch—200 °C, temperature of environment—30 °C. The characteristics for the forging aggregate—a MaxiPress press—were assumed from the program’s database. The Tresca friction model was used with friction factor = 0.4. The thermal conductivity coefficient in the contact was 10 kW/m2·°K, and the thermal conductivity coefficient with the environment was 15 W/m2·°K. All the tools were assumed to be non-deformable with a heat exchange. Steel 1.4981 was selected as the forging material from the program’s database because it demonstrates similar mechanical properties to those of the charge material used in the real process. The tool material was steel grade 1.2343, and its properties were adopted from the simulation software’s material database. Figure 14 presents exemplary results with fields of deformation and temperature on the forging at the last forging stage. You can observe the biggest plastic deformations in the “leg” of the forging, which equal about 2. The case is similar in the temperature field distribution. This said, we can see that, as a result of the change of plastic deformation work into heat and friction, in the areas of the forging’s leg, the temperature increased from the initial temperature, 1050 °C, to 1100 °C. In the remaining areas the temperature decreased at the surface layer, even to 150 °C, up to 850 °C as a result of the contact forging with the cooler forming tools.

**Figure 15:**
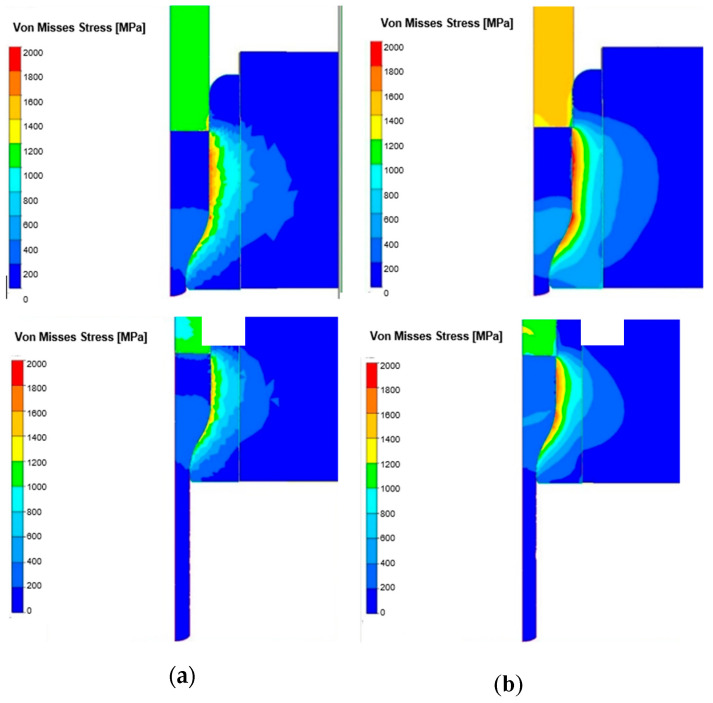
FEM results with distributions of von Misses pressures at the start and finish of the extrusion process: (**a**) standard process, (**b**) for a bigger hardness of the input material.

In Figure 15 we can observe a comparison of the von Misses pressure distributions at the start and finish of the extrusion process for both options. The analysis of the results for both process options showed that the highest stress takes place in the upper cylindrical area of the die and at the beginning of the cross-section reduction. 

**Figure 16:**
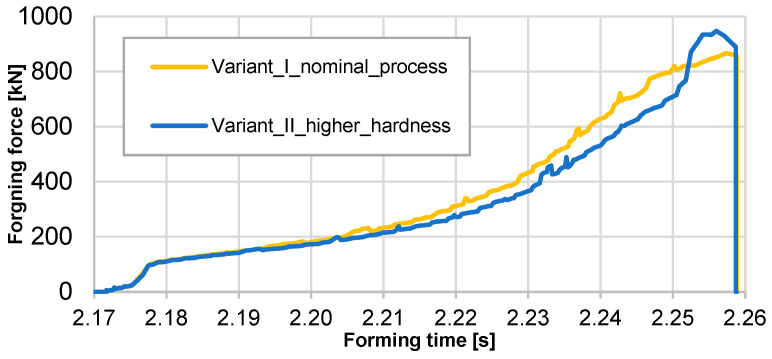
FEM results with extrusion forces.

As expected, for higher hardness of the input material, the von Misses pressures reached as much as 1800 MPa, whereas those for the standard process (hardness of input about 200 HV) reached about 1400 MPa. In the end of the extrusion process for both options, the pressures were smaller but still bigger and equal: 1100–1200 MPa in the standard process and 1500–1600 MPa for input material with higher hardness. In Figure 16 we can see extrusion forces in the function of forming time.

**Figure 17:**
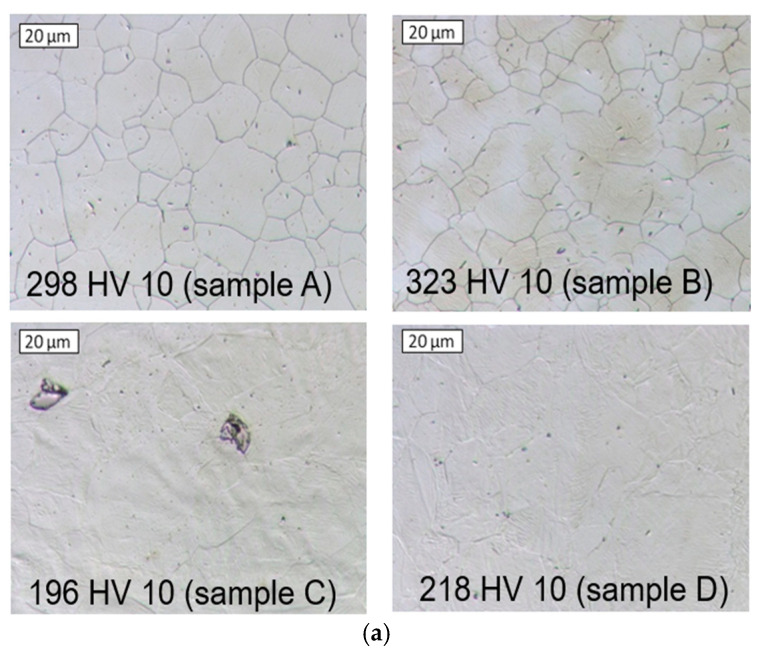

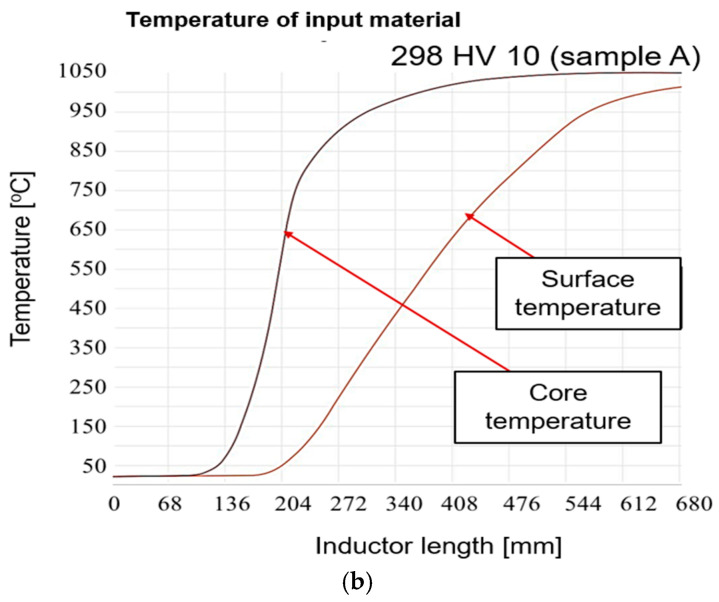
The results: (**a**) result of microstructural tests for selected charges (input material) with hardness after heating (298, 323, 196, 218 HV); (**b**) temperature distribution for heating of an example input material.

For input material with higher hardness, the maximal values of force are bigger by about 80–100 kN in relation to the standard process. In addition, in the case of the harder material, the extrusion force increases faster in the final phase of process. At the following stage of the research, tests of a few randomly selected forgings were conducted after single heating and cooling in water (collected at distances of 50 inputs). In Figure 17 is shown microstructure photos of the charge materials for a few randomly selected input materials with the lowest and highest hardness and temperature distribution for heating of an example input material.

**Figure 18:**
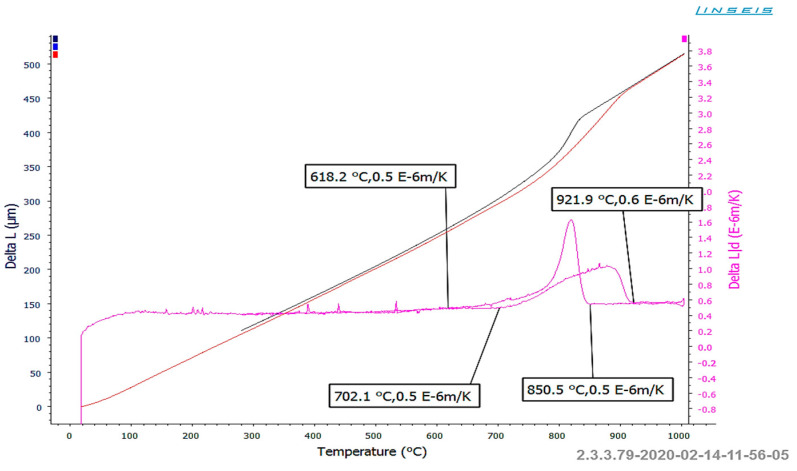
The dilatometric curve for the NC3015 material (steel) [31].

The microstructural analyses revealed that the samples subjected to heating exhibited significant variability in hardness values. This variability is attributed to the different degrees of dissolution of hard carbide precipitates at the grain boundaries in the input material. Despite using consistent heating parameters for each sample, achieving complete dissolution of the carbides in these regions was not possible. Consequently, the material after supersaturation displayed a range of hardness from 196 to as high as 323 HV10. To identify the temperature ranges necessary for dissolving hard carbide phases at the grain boundaries in austenite, specific tests and dilatometric studies were conducted. The samples were carefully prepared and heated to 1060 °C at a rate of 6 °C/min, followed by cooling at the same rate to approximately 180 °C. It is important to note that this heating rate is significantly lower than that used in industrial settings due to the limitations of the dilatometer. Figure 18 illustrates the baseline and the first derivative obtained from the thermal analysis conducted.

Taking into account the analyzed issue, it is important to consider the curves obtained during heating. The course of the diagrams makes it possible to state that during the heating and cooling of the alloy, transformations take place, which can be connected with precipitation and dissolution of a number of phases at the grain boundaries. The initiation of dissolution of these phases during the alloy’s heating takes place at about 700 °C, and the process lasts to a temperature of over 920 °C. This means that at the typical industrial heating temperature, the carbide phases located along the prior austenite grain boundaries should begin to dissolve. In addition, it should be noted that this process is distributed over a wide temperature range, which causes the most intensive dissolution of carbides to occur at the highest temperatures. On the basis of the performed complex analysis aided with numerical modeling, it was established that, with the aim to ensure repeatability of the heating process, it is necessary to reconstruct the induction heater by way of adding an additional zone of charge material heating in the final heating phase. The preliminary research showed that such additional heating of the charge causes almost total dissolution of the carbides and thus also solves the problem of premature wear of the die as a result of too high hardness of the charge material.

## 4. Summary and Conclusions

This work presents the results of many years of scientific research by the authors on the possibility of using many methods and techniques supported by computer methods, which can be especially used to analyze temperatures and temperature field distribution in a few issues of industrial forging processes. 

The conducted research confirms that precise control of temperature and its distribution on both the billet and the tools is crucial for the forging process, tool durability, and the prevention of adverse phenomena, such as adhesion or billet jamming. The results clearly indicate that even minor measurement errors or local temperature deviations can lead to premature tool failures and disruptions in the technological process. Currently, the most frequently used methods of measuring forgings and forging tools are relatively convenient, non-contact thermographic measurements, supported and verified by contact measurements using thermocouples, as well as the use of numerical modeling as a virtual experiment. Numerical modeling is still the most frequently used tool for this purpose, which owes its popularity mainly to its ease of use and quick and relatively accurate results confirmed in real conditions. The use of numerical programs based on FEM allows for carrying out not only relatively simple global analyses of forging processes to analyze key technological parameters but also measurements and analyses of process correctness to determine key technological parameters, such as distributions of temperatures. This paper also presents the possibility of analyzing temperature changes using both numerical simulations and typical thermal imaging, which is crucial from a technological point of view, as well as the interconnection and complementarity of standard temperature measurement techniques. Studies of changes and distributions of the temperature field were used to analyze forging tools and input material, the so-called steady state using FEM, and the entire technological sequence for selected processes, as well as to assess the overheating of robot grippers. The FEM results were used to analyze the temperature of the input material and the distribution of field temperature of forging tools in each step of the forging process (heat-up of charge, tools, during forging, and also after forging).

A detailed analysis was performed of the process of extrusion of a valve forging, in which premature wear of the tools took place as a result of blocking of one of the first forgings. The conducted investigations demonstrated that a probable cause of such a state of affairs is the lack of total dissolution of the hard M_23_C_6_ carbides during the induction heating of the charge material, which was confirmed by the results of numerical simulations, as well as microstructure tests aided by dilatometric studies. A solution to this problem was a prolongation of the heating time in the final heating phase, which, consequently, was ensured through the development of a new heating system. Based on the presented research results, it can be concluded that commonly used and available measuring devices, such as thermocouples, pyrometers, and thermal imaging cameras, can be used to measure and analyze temperature changes in industrial forging processes. Another good direction is the use of computer simulations using various computational packages due to the relative speed and ease of analysis. The key here is the selection of the appropriate measurement method for a given issue and awareness of its potential advantages and disadvantages, as well as their consequences, i.e., measurement issues. After all, not every issue or phenomenon related to temperature can be measured using all methods. Studies have shown that the best and most reliable measurement results can be obtained by using several measurement methods simultaneously, where one method can be verified by another, which is more suitable for measuring temperature for a given issue. Furthermore, further research and work related to the development of current measurement methods, as well as the search for new ones, in particular, those dedicated to die forging processes at elevated temperatures, are still necessary.

### The Main Conclusions

Effective temperature monitoring in hot forging processes is essential for ensuring tool durability and process stability. The conducted analyses confirmed that even minor inaccuracies in temperature assessment may lead to critical failures, such as adhesion, billet jamming, and premature tool wear.

Among the available methods, non-contact thermographic techniques, when supported by contact measurements (e.g., thermocouples), offer convenient and reliable temperature control under industrial conditions. However, their limitations, such as sensitivity to surface conditions or optical interference, require complementary approaches.

Finite Element Method (FEM)-based numerical modeling remains a key diagnostic and predictive tool in forging technology. It enables both global and local analyses of thermal behavior throughout the entire technological sequence—from billet and tool heating, through deformation stages, to post-forging cooling—and supports optimization of process parameters.

The results show that the most reliable measurement outcomes are achieved through the integration of multiple methods, where numerical simulations are validated with experimental data. This multi-method approach allows not only for better understanding of heat transfer phenomena but also supports the development of practical solutions, such as modified heating schedules or improved tool design, aimed at increasing process reliability.

A case study involving valve extrusion demonstrated that incomplete dissolution of hard M23C6 carbides during induction heating contributed to tool failure. This insight led to the implementation of a revised heating strategy, illustrating the practical impact of integrated temperature analysis on real-life forging operations.

Further research is needed to improve the precision and robustness of measurement techniques under extreme conditions, as well as to develop new methods specifically adapted to the needs of hot die forging applications.

## Figures and Tables

**Table 1:** Chemical composition of analyzed materials.

Element	C [%]	Si [%]	Mn [%]	Cr [%]	Mo [%]	V [%]	P [%]	S [%]	Ni [%]
16MnCrS5 (1.7131)	0.14–0.19	≤0.40	1.00–1.30	0.80–1.10	-	0.10–0.25	≤0.025	≤0.035	–
NCF3015 (1.4016/1.4057)	≤0.08	≤0.50	≤0.50	13.5–15.5	0.40–1.00	-	≤0.015	≤0.010	30.0–33.5
1.2343 (H11)	0.33–0.42	0.80–1.20	0.25–0.50	4.80–5.50	1.10–1.50	0.25–0.50	≤0.03	≤0.03	
1.2344 (H13)	0.37–0.43	0.90–1.20	0.30–0.50	4.80–5.50	1.20–1.50	0.90–1.10	≤0.03	≤0.03	
1.2365 (WLV/H10)	0.28–0.35	0.10–0.40	0.15–0.45	2.70–3.20	2.50–3.00	0.40–0.70			
Unimax	0.50	0.20	0.50	5.00	2.30	0.50			
W360	0.50	0.25	0.50	4.50	3.00	0.60			

**Table 2:** The results of temperature measurements.

Temperature Set on the Induction Heater [°C]	Temperature from Thermovision Camera [°C]	Temperature of Pyrometer on Induction Heater [°C]
1250	1209	1257
	1213	1266
	1197	1259
	1199	1313

## Data Availability

The original contributions presented in this study are included in the article. Further inquiries can be directed to the corresponding author.
